# 

**Published:** 1894-05

**Authors:** 


Civil Service Examination for Women Physicians.—An open competitive examination for the position of woman physician in the state hospitals, will be held at the office of the State Civil Service Commission, Albany, Wednesday, May 23, 1894, at 9 o'clock A. M.

Applicants must be residents of the State of New York, graduates of a legally incorporated medical college, and must have had one year's experience in a hospital, or three years' experience in the general practice of medicine. Limits of age, 21 to 50. Salary, $1,200 per annum and maintenance.

For application blank, address New York Civil Service Commission, Albany, N. Y.

THOMAS CARMODY,
*Chief Examiner.*

Albany, N. Y., April 13, 1894.

The *New York State Medical Reporter* made its appearance in March, 1894. It is a monthly journal of medicine and surgery, edited by H. Bronson Gee, M. D., and published at Rochester, N. Y. It is a handsomely printed double-columned magazine of thirty-two pages, imperial octavo in size, and judged by its first number it will become a valuable addition to periodical medical literature. Its initial number contains an article by Dr. Louis A. Weigel, of Rochester, on the orthopedic treatment of deformities of paralytic origin, that is well written and handsomely illustrated.

---

THE Plimpton Manufacturing Co., of Hartford, Conn., publish in convenient form, a Physicians' Bedside Record with Dietary, edited by Gideon C. Segur, M. D. Each book is designed for use but in a single case, and the physician writes his directions for the treatment of the patient at the bottom of the page each day. The nurse is to record each and every event connected with the patient at the time of its occurrence. The price of this record is ten cents each, or one dollar per dozen, to be had on application to the publishers.

---

THE REFRACTIONIST, a journal of practical ophthalmology, intended to be an exponent of the refraction world, has just appeared. Published monthly. Editor, Francis F. Whittier, A. M., M. D., Professor Clinical Ophthalmology, College of Physicians and Surgeons; Ophthalmic Surgeon St. Elizabeth Hospital; formerly on Resident Staff Manhattan Eye and Ear Hospital, etc., 74 Boylston street, Boston. With associate editors. Subscription price, $2 yearly.

---

LISTOL CHEMICAL COMPANY, of Chicago, offer to the profession a new chemical compound of thymol and iodine, called listol, for surgical purposes. It is claimed to be a valuable surgical dressing in surgery, dentistry, bed sores, burns and all erosions of the skin or mucous membrane. Their advertisement will be found on page xxxiii. of this issue.

---

MESSRS. O. W. CLARK & SON, of 59 Seneca street, Buffalo, have sent out their annual catalogue for 1894. It is a handsomely illustrated paper of forty-eight pages, with illuminated cover, that tells all about farm, field, vegetable and flower seeds, and we advise those who need any of these to consult the Messrs. Clark.

---

TAYLOR BROS. COMPANY, of Rochester, N. Y., appear in our advertising columns on page xi., offering to the profession something of great usefulness and value.

